# Meteorin-Like Shows Unique Expression Pattern in Bone and Its Overexpression Inhibits Osteoblast Differentiation

**DOI:** 10.1371/journal.pone.0164446

**Published:** 2016-10-07

**Authors:** Weiyan Gong, Yong Liu, Zhihong Wu, Shaohai Wang, Guixing Qiu, Shouqing Lin

**Affiliations:** 1 Department of Obstetrics and Gynecology, Shandong Provincial Hospital affiliated to Shandong University, Jinan, China; 2 Department of Orthopaedics, Peking Union Medical College Hospital, Chinese Academy of Medical Sciences & Peking Union Medical College, Beijing, China; 3 Department of Obstetrics and Gynecology, Peking Union Medical College Hospital, Chinese Academy of Medical Sciences & Peking Union Medical College, Beijing, China; Kyungpook National University School of Medicine, REPUBLIC OF KOREA

## Abstract

The present study was performed to identify and characterize genes involved in osteoblasts function. Firstly, we constructed and sequenced a human osteoblast full-length cDNA library to screen for genes whose functions have not been reported and further identify these candidate genes through detecting the relationship with the activator protein-1 (AP-1) transcription factor complex using a dual luciferase reporter system. Only one gene, namely METRNL (Meteorin, glial cell differentiation regulator-like) has been screened out. We performed immunohistochemistry to analyze expression patterns in bone and established a stable transfection MG63 cell line of METRNL-EGFP fusion protein overexpression to analyze the function of METRNL in mineralized nodule formation. Immunohistochemistry showed METRNL expression in hypertrophic chondrocytes and osteoblasts lining trabecular bone surfaces. Overexpression of METRNL inhibited mineralized nodule formation by the MG63 osteosarcoma cell line. Thus, the identified gene, METRNL, which is associated with AP-1 transcription factor complex activity, has a unique expression pattern in bone. In addition, the anomalous expression of METRNL may inhibit bone cell differentiation.

## Introduction

Osteoblasts originate from pluripotent precursors that are also capable of differentiating into adipocyte, chondrocyte, and muscle lineages[1]. Differentiation of osteoblasts is a complex and highly coordinated process[2]; many factors participate in this process. In recent years, several signaling pathways and transcription factors that control osteoblast differentiation and function have been elucidated; however, the functions of many osteoblast-expressed proteins remain unknown. With the aim of identifying new genes involved in osteoblast proliferation, differentiation, and function, we first constructed and sequenced a human osteoblast full-length cDNA library[3]. Then, genes for which no function has been reported in osteoblasts were chosen for analysis.

As described previously, members of the activator protein-1 (AP-1) transcription factors play an important role in bone cell proliferation and differentiation. The AP-1 transcription factor complex consists of a large variety of dimers formed by members of the Fos, Jun, and activating transcription factor (ATF) families[4]. Overexpression of ΔFosB or Fra-1 in transgenic mice increases bone formation[5,6]. Additionally, AP-1 activity also participates in the function of transformation growth factor β[7], parathyroid hormone[8], and 1,25-dihydroxy vitamin D in osteoblasts [9]. Thus, detection of the activity of AP-1 transcription factor complex was used as a tool for further identifying the genes involved in osteoblast function using a dual luciferase reporter gene assay system. Then the open reading frame (ORF) of each candidate gene was subcloned into the pcDNA3.1 vector, and cotransfection with pAP1-luc/pRL-TK into 293T cells. Furthermore, the identified genes were analyzed on the basis of immunohistochemical findings and mineralization function in MG63 cells.

After screening from the human osteoblast full-length cDNA library, the candidate genes, whose functions have not been reported in osteoblasts before, have been identified. Based on the relationship with AP-1 transcription factor complex, only one gene, namely METRNL, has been screened out. Additionally, our preliminary study showed that METRNL is specially expressed in bone and overexpression of METRNL inhibited mineralized nodule formation by the MG63 osteosarcoma cell line.

## Materials and Methods

### Ethics Statement

All animals were handled according to the Standards for Laboratory Animals (GB14925-2001) established by the People’s Republic of China. All animal procedures were approved by the Institutional Animal Care and Use Committee of Peking Union Medical College Hospital (approval number: SYXK 2005–0008). The animals were euthanised via decapitation under sodium pentobarbital anesthesia, and all efforts were made to minimize suffering.

### Cell Culture

MG63 (human osteosarcoma cells), CHO, and 293T cells were purchased from the Cell Resource Center of the Institute of Basic Medical Sciences, Chinese Academy of Medical Sciences. All cells were cultured at 37°C, 5% CO_2_ in Dulbecco’s Modified Eagle Medium (DMEM) supplemented with 10% fetal bovine serum and 1% penicillin/streptomycin.

### Cloning and Vector Construction

Total RNA was isolated from MG63 cells with Trizol reagent and reverse-transcribed with Superscript^TM^ II Reverse Transcriptase (Invitrogen, Carlsbad, CA, USA). The full-length predicted ORF of METRNL was amplified by Herculase polymerase (Stratagene, San Diego, CA) with a P1 primer pair (Table 1), and 1.5 μl DMSO in a 50-μl reaction volume. Cycling conditions were initial denaturation at 98°C for 3 min, 30 cycles of denaturation at 98°C for 20 s, annealing at 56°C for 20 s, and extension at 72°C for 40 s, followed by a final extension for 5 min at 72°C. METRNL was cloned into the *Hind*III and *BamH*I sites of pcDNA3.1 (+).

**Table 1:** Sequences of primers used for gene cloning and quantification of gene expression by quantitative real-time PCR.

Primer name	Sequence (5′-3′)
P1 sense	GAGCCGGCGCCAGAGCATG
P1 anti-sense	GGAGTCAGTCCGTGCCAACCT
P2 sense	CCCAAGCTTGAGCCGGCGCCAGAGCATG
P2 anti-sense	CGGGATCCAGGCAGTCCGTGCCAACCT
P3 sense	GCGAATTCATGTACCCAACAGGTGCTCTC
P3 anti-sense	CCCAAGCTTGTCCGTGCCAACCTCACAAG
Osteocalcin sense	ATGAGAGCCCTCACACTCCTC
Osteocalcin anti-sense	GCCGTAGAAGCGCCGATAGGC
METRNL sense	CAAGTTACCCACGAGCCTGAG
METRNL anti-sense	CGGCCCACGGAGTCAGTC
OPG sense	CCCTGTGTGAGGAGGCATTCTTCA
OPG anti-sense	CACTCTCTGCGTTTACTTTGGTGCCA
GAPDH sense	ATTGTTGCCATCAATGACCC
GAPDH anti-sense	AGTAGAGGCAGGGATGATGT

We generated an METRNL-EGFP fusion by amplifying the METRNL gene with mutagenic primers P2, and then cloned the amplified fragment into pEGFP-N1 to construct the pMETRNL-EGFP-N1 vector. We used the same method to generate full-length myc/6× his-tagged METRNL by subcloning the gene fragment into pcDNA4-myc/his. The cDNA sequence encoding METRNL aa 83–311 was amplified with primers P3 and cloned into pET28a. The resultant fusion protein was purified and used to immunize rabbits.

All plasmids were confirmed by DNA sequencing on an ABI 3771 DNA sequencer.

### Reporter Gene Assays

A dual-luciferase reporter system was used to screen for genes involved in AP-1 signaling. Briefly, 293T cells were seeded in a 96-well plate (1.2 × 10^4^ cells/well), incubated for 24 h, and co-transfected with 40 ng pAP-1-Luc (contains seven AP-1 binding elements upstream of the promoter region that drives expression of firefly luciferase), 50 ng pcDNA3.1-METRNL (or pcDNA3.1 vector control), and 4 ng pRL-TK (encoding TK promoter-driven Renilla luciferase). Eighteen hours after transfection, the cells were lysed with Passive Lysis Buffer (Promega, Madison, Wis). Dual-luciferase assay reagent ((Promega, Madison, Wis) was added to 10 wells to very luciferase activity on a Tecan luminometer. AP-1 was stimulated with PMA (50 ng/mL) and ionomycin (50 ng/mL) 18–24 h after co-transfection and luciferase activity was assessed after 6 h. Each experiment was performed in triplicate and data are expressed as the means after normalization for Renilla luciferase activity.

### Rabbit Anti-human METRNL Polyclonal Antibody Production

pET28a-METRNL (88–311) was transfected into BL21 CodonPlus, and then induced for 2 h with IPTG. The cells were lysed and the METRNL-his fusion protein was purified on a Ni-NTA column (Qiagen, Hilden, Germany). The purified protein was mixed with complete Freund’s adjuvant and injected into rabbits to produce rabbit polyclonal antibodies against human METRNL (88–311).

### Stable Transfected Cell Lines

The expression vector pcDNA4-myc/his-metrnl was introduced into CHO cells by transfection. After 24 h, the cells were divided 1:10 into a 10-cm culture plate. At 48 h after transfection, the stably transfected cells were selected by growth in a complete medium containing 800 μg/mL G418. Two weeks later, single clones were screened and cultured in a complete medium containing 400 μg/mL G418. These two cell lines were used to detect the METRNL in supernatant.

Using the same method, the expression vector pMETRNL-EGFP-N1/pEGFP-N1 was introduced into MG63 cells and the overexpressing METRNL-EGFP and EGFP MG63 cell lines were established. Two weeks later, single colonies were screened for fluorescence intensity under a fluorescent microscope and cultured in a complete medium containing 400 μg/mL G418.

### Protein Extraction and Western Blotting Analysis

CHO cells overexpressing METRNL-myc/6× his were seeded in 6-well plates containing the complete culture medium. When the cells reached 80% confluence, they were washed and cultured in serum-free DMEM medium for 24 h. The supernatant was collected and ultra-filtered through a 10-kDa cut-off membrane (Millipore Amicon Ultra15 Centrifugal filter Units) at 5000 × *g* for 30 min. The recombinant METRNL-myc/6× his was purified with Talon metal affinity resin (Clontech, Palo Alto, CA). The purified protein and whole cell lysates of CHO cell and CHO cells overexpressing METRNL were analyzed by western blotting with rabbit anti-METRNL (88–311) polyclonal antibody and peroxidase conjugated goat anti-rabbit IgG (1:1000, Santa Cruz Biotech, Santa Cruz, CA).

### Histology and Immunolocalization

Three-week-old female Sprague-Dawley rats were anesthetized and sacrificed and the tibias dissected for analysis. The tibias were fixed in neutral buffered formalin, decalcified in 14.5% buffered ethylenediamine tetra-acetate, washed in PBS, and embedded in paraffin wax. Serial sections were cut, deparaffinized in two changes of xylene (10 min), and rehydrated through descending concentrations of alcohol prior to hematoxylin-eosin and immunohistochemical staining. For immunohistochemistry[10], decalcified tibial sections were stained with METRNL polyclonal antibody or pre-immune rabbit serum for 1 h at 37°C followed by biotinylated goat anti-rabbit IgG and avidin-biotin-peroxidase.

### Induction of Mineralization and von Kossa Staining

MG63 cells overexpressing EGFP and METRNL-EGFP grown to 80%–90% confluence were treated with β-glycerophosphate (10 mmol/L), ascorbic acid (50 μg/mL), and dexamethasone (1 × 10^−8^ mol/L) for 21 days. Then the cells were fixed with 95% ethanol for 10 min, washed three times with distilled water, and incubated with 5% silver nitrate solution ultraviolet (UV) for 1 h, and then washed with distilled water for three times and treated with 5% sodium thiosulfate. Calcium particles were observed at 100× magnification.

### Real-time PCR

MG63 cells overexpressing EGFP and METRNL-EGFP cultured in with β-glycerophosphate (10 mmol/L), ascorbic acid (50 μg/mL), and dexamethasone (1 × 10^−8^ mol/L) for 7 days and 14 days. Total RNA was isolated from MG63 cells with Trizol reagent and reverse-transcribed with Superscript^TM^ II Reverse Transcriptase (Invitrogen, Carlsbad, CA, USA). Real-time PCR was performed on a Rotor-Gene 2000 (Corbett Research), each reactions contained 10 μL of QuantiTect SYBR Green PCR Master Mix (Qiagen, Hilden, Germany); 0.5 μM specific primers (Table 1) and 30 ng of cDNA template. Messenger RNA (mRNA) expression was calculated using the ΔΔCt method and normalized to the expression of GAPDH. Primers used are outlined in Table 1.

### Statistical Analyses

Results were expressed as means ± standard deviations. Student’s t-test was used for statistical comparison. P < 0.05 was considered statistically significant.

## Results

### Changes in METRNL expression suppresses AP-1 transcription factor complex activity

**Fig 1:**
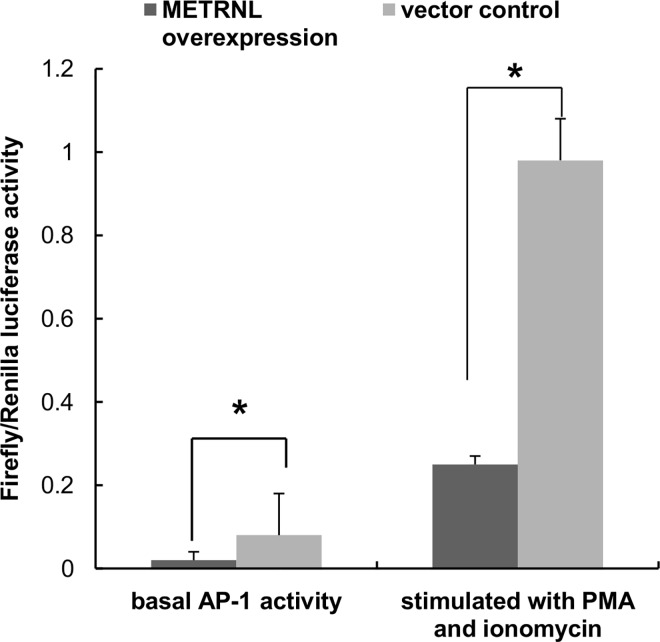
Overexpression of METRNL suppresses AP-1 transcription factor complex activity in 293T cells. In cells co-transfected with pCDNA3.1-METRNL, pAP-1, and pRL-TK, METRNL suppressed basal endogenous AP-1 transcription factor complex activity. METRNL also inhibited AP-1 transcription factor complex activation in cells treated with exogenous AP-1 activity stimulator (PMA and ionomycin). Data showing means from three independent experiments are presented after normalization for Renilla luciferase activity. *P < 0.01

After screening from the human osteoblast full-length cDNA library, fifteen genes for which no function has been reported in osteoblasts have been identified. Then activity of AP-1 transcription factor complex has been also detected to further identify the genes involved in osteoblasts function. The open reading frames (ORFs) of these candidate genes including METRNL were subcloned into the pCDNA3.1 vector and cotransfected with pAP1-luc/pRL-TK into 293T cells. Only overexpression of METRNL significantly reduced basal activity, which was reduced by 73.86% (Fig 1). Data for other candidate genes which did not affect AP-1 transcription factor complex activity were not shown.

To verify this inhibitory function, we treated the cells with PMA (50 ng/mL) and ionomycin (50 ng/mL) 18–24 h after co-transfection to stimulate AP-1 transcription factor complex activity. Reporter gene assays indicated that PMA- and ionomycin-induced AP-1 transcription factor complex activation was significantly down-regulated in pCDNA3.1-METRNL transfected cells in comparison to that in empty control vector-transfected cells (Fig 1).

### METRNL Shows Unique Expression Pattern in Bone

**Fig 2:**
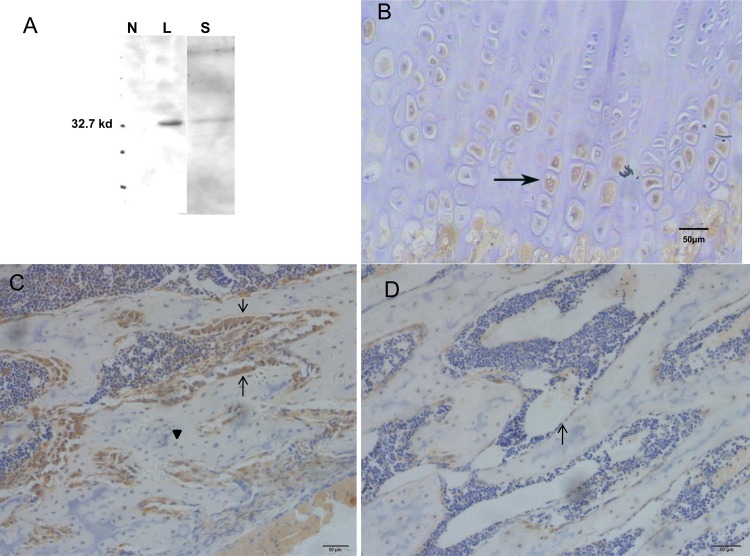
Immunohistochemistry for METRNL protein in rat bone. (A) In western blotting analyses, METRNL expression is observed in lysates (L) and supernatants (S) of stable CHO cells overexpressing His-tagged METRNL, indicating that METRNL indeed is a secreted protein and the polyclonal antibody was specific to METRNL (N for lysates of CHO cells). (B) METRNL is detected in hypertrophic chondrocytes (→) in the tibial growth plate of three-week-old rats. (C) In the primary spongiosa, METRNL is detected in osteoblasts (→) lining the trabecular bone surfaces, but osteocytes (▼) are absent.(D) In the secondary spongiosa, METRNL protein is detected weakly and diffusely in osteoblasts lining the bone surfaces (→).

After the primary screening step, we focused on the expression and function of METRNL in bone. We purified METRNL (88-311aa)-his fusion protein, and produced rabbit anti-METRNL (88–311) polyclonal antibody. In western blotting analyses with this antibody, stable CHO cells overexpressing His-tagged METRNL showed expression in the lysates and culture supernatants (Fig 2A), indicating that METRNL indeed encodes a secreted protein and our polyclonal antibody was specific to METRNL.

METRNL expression in the tibias of 3-week-old female rats was determined by immunohistochemistry. METRNL immunoreactivity was mainly detected in hypertrophic chondrocytes, but resting and proliferative chondrocytes were negative for METRNL expression in the tibial growth plates (Fig 2B). Staining was confined to cytoplasmic granules. Intense METRNL staining was also observed in active osteoblasts lining the trabecular bone surfaces and negative staining was observed in osteocytes (Fig 2C). In secondary spongiosa, the osteoblast is not actively synthesizing bone, the surface osteoblasts are called inactive osteoblasts. METRNL protein was detected weakly (Fig 2D).

### Overexpression of METRNL Inhibited the Differentiation of MG63

**Fig 3:**
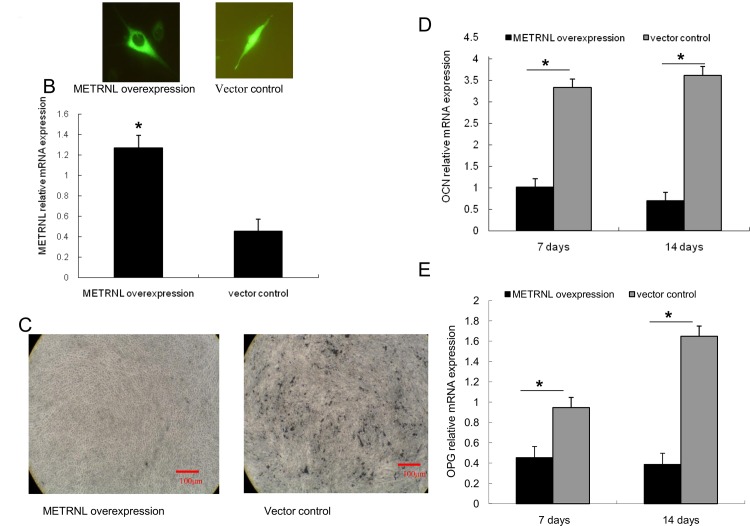
Stable overexpression of METRNL may inhibit MG63 cell differentiation. (A) METRNL-EGFP fusion protein was highly expressed in the cytoplasm of MG63 cells. (B) Real-time PCR data suggest that METRNL mRNA levels in the METRNL overexpression group were significantly higher than those in the control. (C) Overexpression of METRNL suppresses mineralized nodule formation in MG63. Mineralized nodule formation is observed in EGFP-overexpressing cells in mineralizing medium. Mineralized nodule formation is absent in cells overexpressing pEGFP-METRNL. (D, E) Quantification of OCN and OPG, detected by RT-PCR after treatment with mineralization condition medium for 7 days and 14 days, revealed remarkable downregulation of these genes in the METRNL overexpression group compared with controls (*P < 0.05).

Osteosarcoma-derived MG63 cells were used to study the effect of METRNL on mineralized nodule formation in vitro. The stable transfection cell line was observed by scanning-laser confocal microscopy. As Fig 3A shows, the METRNL-EGFP fusion protein was highly expressed in cytoplasm of MG63 cells, and the real-time PCR data suggest that METRNL mRNA levels (including the METRNL and METRNL-EGFP fusion protein) were higher than control (Fig 3B).

After culture in a mineralization condition medium for 21 days, mineralized nodules were stained by von Kossa. Mineralized nodule formation was observed in EGFP-overexpressing cells in the mineralizing medium, but mineralization was completely absent in cells overexpressing METRNL-EGFP (Fig 3C).

We used real-time-PCR to detect OPG and OCN mRNA expression in the two groups after they had been treated with mineralization condition medium for 7 days and 14 days (Fig 3D and 3E). Quantification of OPG and OCN revealed a remarkable down-regulation of these genes in the METRNL overexpression group compared with controls (P < 0.05).

## Discussion

Osteoblasts proliferation and differentiation are regulated by multiple factors. Although several genes and pathways have been identified to be involved in osteoblasts proliferation and differentiation, lots of potential genes have not been fully elucidated. Therefore, we constructed and sequenced a human osteoblast full-length cDNA library and identified the candidate genes whose functions have not been reported in osteoblasts before. A total of 15 genes were identified. To further screen for the genes involved in osteoblasts function, association of candidate genes expression with AP-1 transcription factor complex activity has been detected using a dual luciferase reporter gene assay system. As it was reported previously, AP-1 has been implicated in a variety of cellular processes including cell proliferation, differentiation, apoptosis, and oncogenic transformation [11,12]. Data from transgenic mice show that some components of AP-1 are also key regulators of bone cell differentiation [5,6]. Thus, identification and characterization of new proteins that affect AP-1 activity will cast new light on the regulation of skeletal development. Following our results, only METRNL was identified to be associated with AP-1 activity. Additionally, the inhibitory effects of AP-1 activity of METRNL have been confirmed by addition of PMA and ionomycin, both of which are exogenous AP-1 activity stimulators. Then we explored the expression pattern in osteoblasts as well as the effects of METRNL on osteoblasts differentiation. METRNL is a secreted protein and has been shown to inhibit mineralized nodule formation as well as the expression of OPG and OCN.

METRNL immunoreactivity was mainly detected in hypertrophic chondrocytes in the tibial growth plate of three-week-old rats. The growth plate is divided into three regions: the resting, proliferative, and hypertrophic zones. Chondrocytes proliferate from the resting zone and then differentiate into hypertrophic chondrocytes, which are actively engaged in cartilage matrix synthesis and secretion.[13]. This process is tightly controlled by a complex network of signaling molecules that exhibit special expression patterns in the growth plate[14]. For example, Indian hedgehog (Ihh) and parathyroid hormone-related protein (PTHrP) participate in the regulation of chondrocyte differentiation. Ihh is synthesized only by early hypertrophic chondrocytes[14,15]. In the postnatal growth plate, fibroblast growth factor receptors (FGFRs) 1 and 3, which inhibit chondrogenesis, are expressed in the proliferative and hypertrophic zones. In contrast, FGFRs 2 and 4, positive regulators, are expressed in the resting and proliferative zones[16]. Moreover, different stages of osteoblasts express different proteins that participate in different functions. Although the biological function of METRNL in bone is unknown, its unique expression pattern in the growth plate and primary spongiosa provides insight into its role in regulating osteoblast and chondrocyte differentiation.

Meteorin has been characterized as a family of neurotrophic factors that promote axon extension and alter satellite glial morphology in sensory ganglia[17]. The N-terminus of METRNL shares homology with that of Meteorin. Recently, METRNL, Dclk1, and Serpinb1a were identified as latent process (LP) genes. The latent process corresponds to a preparation step for neurite extension. Co-overexpression of all LP genes enhances neurite extension[18]. In the growth plate, after differentiating into hypertrophic chondrocytes, chondrocytes stop division and actively engage in cartilage matrix synthesis and secretion and produce factors that stimulate vessel invasion[13]. We suggest METRNL functions as a latent process gene in hypertrophic chondrocytes, preparing the cells for further function, although its mechanism requires further study.

In vitro, osteoblast differentiation is characterized by three principal periods: proliferation, extracellular matrix production, and mineralization[19]. Terminal differentiation of osteoblasts in vitro is characterized by the formation of mineralizing nodules. MG63 cells are widely used as a model for human osteoblasts. These cells display several osteoblastic traits that are typical of a relatively immature osteoblast and can be induced to mineralize by adding ascorbate-2-phosphate, β-glycerophosphate, and dexamethasone[20]. In our study, control cells overexpressing EGFP formed mineralizing nodules as detected by von Kossa staining after 21 days induction, consistent with previous reports[20,21]. MG63 cells overexpressing METRNL-EGFP did not exhibit matrix mineralization.

The main limitation in the present study is the limited knowledge of the relationship between METRNL and AP-1 transcription factor complex. We just tested whether changes in expression of candidate genes influence the activity of AP-1 transcription factor complex and the mechanism of this interaction such as which AP-1 dimers are activated has not been explained. Further study is still needed to explore the underlying mechanism of METRNL participating in AP-1 transcription factor complex mediated osteoblast function.

In conclusion, after screening from a human osteoblast full-length cDNA library and further identifying based on the relationship with AP-1 transcription factor complex, only one gene, namely METRNL that was supposed to be involved in osteoblast function, has been screened out. METRNL is a secreted protein and mainly expressed in hypertrophic chondrocytes of the growth plate and in osteoblasts lining trabecular bone surfaces. Overexpression of METRNL inhibited mineralized nodule formation by MG63. Further studies are needed to investigate the physiological role of METRNL in bone.
